# Plate Waves Scattering Analysis and Active Damage Detection

**DOI:** 10.3390/s21165458

**Published:** 2021-08-13

**Authors:** Tai-Ho Yu

**Affiliations:** Department of Electronic Engineering, National United University, 2 Lien Da, Nan-Shih Li, Miaoli 36063, Taiwan; yth@nuu.edu.tw

**Keywords:** plate waves, wavelet transform, time-of-flight difference, simplex algorithm

## Abstract

This study investigates and evaluates the technology of using plate waves to detect the locations and sizes of circular holes and cracks in plates. Piezoelectric ceramic discs surface-mounted on both sides of an aluminum alloy plate were used as narrow-frequency plate wave actuators and sensors, and the antisymmetric plate wave signal was analyzed by wavelet transform in the time-frequency domain. The damage location and frequency spectrum characteristics were identified by the wave through time-of-flight difference and signal analysis of the damage scattered wave group. The plate wave signal of the damaged plate included the scattered wave signal and the plate wave signal transmitted directly between the piezoelectric discs. Under ideal conditions, the plate wave signal indicating damage can be obtained by subtracting the plate wave signal in a plate without damage from the plate wave signal scattered from actuators to sensors. This study established an optimization program based on the simplex algorithm to inversely calculate the location of the plate damage. The developed damage location objective function has a unique global minimum value that can ensure the accuracy of the damage location calculation, and good results were obtained in experiments. The spectral characteristics of the scattered plate wave were related to the type, size, wave propagation path, and incident angle of the damage. Numerical analyses of scattered spectra for various damages are needed as references to compare with experimental results in the future.

## 1. Introduction

Piezoelectric materials have the advantages of light weight and small size, and they are easy to adhere to the surface of a structure or embed in the structure. When the piezoelectric element shrinks or expands in the transverse direction, the structure is flexural deformed, and a plate wave is generated.

An ultrasonic detection transducer is usually composed of piezoelectric materials that can greatly reduce the time and labor required for traditional testing of network communication technology. These materials can also be developed and integrated into a “structural health monitoring” system.

Degertekin et al. [1] designed a contact transducer with Hertzian contact force in 1996, and successfully excited a Lamb wave on an aluminum plate. The transducer could be designed according to the different shapes of quartz rods, giving the aluminum plate a different contact force. The transducer could excite *A*_0_ and *S*_0_ plate waves or single-mode plate waves and had a high signal and noise ratio (SNR) value. In the same year, Degertekin et al. [2] used a Hertzian contact transducer to excite an *A*_0_ plate wave on an anisotropic plate to obtain the phase velocity dispersion curve of *A*_0_ and examine the effect of delamination on the phase velocity. Good consistency was obtained in theoretical models and experimental measurements.

Banks et al. [3] attached piezoelectric materials to both sides of aluminum alloy cantilevered beams in 1996. The material properties of the beams included mass density and elastic modulus. The change of damping coefficients was combined with a Galerkin numerical solution to estimate the size and location of the damage. Moulin et al. [4] discussed the feasibility of embedding PZT piezoelectric materials in composite plates to excite multi-mode Lamb waves. The waves were simulated by the finite element method, and the obtained modal analysis was in good agreement with the experimental measurement results. In the same year, Chang et al. [5] attached PZT piezoelectric ceramic elements to a fiber-wound composite tube to form a set of arrays. The piezoelectric elements not only acted as a transmitter of flexural waves but also could receive flexural waves.

Roh [6] also attached a PZT piezoelectric ceramic element to an aluminum alloy plate in 1998 to form a set of arrays as the transmitter and receiver of the flexural wave and used short-time Fourier transform to calculate the spectral characteristics of the scattered wave of the circular hole and estimate the location and size of the circular hole by the least square difference method.

Paget et al. [7] discussed embedding PZT piezoelectric materials in the midplane of carbon fiber composite plates in 1999 to detect the changes in the strength of composite plates. Jiang et al. [8] also attached piezoelectric materials to aluminum alloy beams in 1999 and simulated damage by clamping small aluminum plates. The wave velocity could be measured from the time-of-flight of longitudinal waves to the cracks. The crack position could be obtained according to the time difference between the longitudinal wave reaching the crack and the receiving point.

Grondel et al. [9] studied the growth of fatigue cracks on aluminum alloy plates containing rivets in 1999. The rivet-containing aluminum alloy plate used piezoelectric materials to generate and receive ultrasonic signals. The signals were analyzed with the Hilbert transform. Gachagan et al. [10] adhered a comb transducer to the surface of a composite plate in 1999 as the emitter of Lamb waves. The optical fiber was attached to the surface and midplane of the composite plate, a He-Ne laser was used as the wave source, a Mach-Zehnder interferometer was used as the receiver of the Lamb wave, and the phase velocity of the Lamb wave was further measured. In 1999, Boller et al. [11] presented a method of monitoring structure damage based on an actuator-sensor system. Seydel et al. [12] proposed a real-time impact identification of stiffened composite panels in the same year.

In 2000, Paget et al. [13] used the PZT piezoelectric material embedded in the midplane of the carbon fiber composite plate to excite a plate wave, used the acoustic emission probe placed on the surface to receive the signal, and transformed the time domain signal into the frequency domain signal by fast Fourier transform. Pai et al. [14] glued PZT devices on a plate and cantilever beam in 2000 as a plate wave actuator and measured the deflection on the plate and cantilever beam with a scanning laser vibrometer. In 2000, Giurgiutiu et al. [15] presented an active sensor for monitoring the health of aging aerospace structures. In the same year, Grondel et al. [16] proposed a study of fatigue cracks in riveted plates by acoustic emission and Lamb wave analysis.

Grondel et al. [17] used the finite element method to simulate the wave propagation behavior of piezoelectric material attached to a composite plate in 2001 and proved that the normal mode of *A*_0_ was greater than S_0_. Kessler [18] researched the frequency response method of passive damage detection and the Lamb wave of active damage detection in 2002. In 2004, Ma et al. [19] proposed applying a smart active sensing technique on damage detection of smart composite plates. In 2005, Giurgiutiu et al. [20] reviewed the state of the art in an emerging new technology, i.e., embedded ultrasonic nondestructive evaluation (NDE).

In 2009, Staszewski et al. [21] developed both an active method and a passive method for impact damage detection. In 2009, Ng et al. [22] presented the application of Lamb waves to inspect damage in composite laminates.

In 2013, Meyers et al. [23] investigated the design and performance of piezoelectric nanocomposite-based interdigitated transducers (IDTs) for active sensing and damage detection. In 2013, Uhl et al. [24] proposed a new method that was a very efficient numerical simulation tool for Lamb wave propagation modeling in aluminum plates exposed to temperature changes. In 2014, Zhong et al. [25] proposed a near-field multiple signal classification (MUSIC) algorithm to detect the damage on aircraft composite structures. In 2015, Rojas et al. [26] presented the implementation of a network of piezoelectric sensors randomly placed on a plate-like structure to detect and locate artificial damage. In 2016, Shen et al. [27] presented a combined analytical finite element model approach for the accurate, efficient, and versatile simulation of 2-D Lamb wave propagation and interaction with damage.

In 2018, Wagner et al. [28] presented highly sensitive electromechanical piezoresistive pressure sensors based on large-area layered PtSe_2_ films. In 2018, Kudela et al. [29] proposed that the piezoelectric transducer arrays be utilized in structural health monitoring systems as a means of excitation and sensing of elastic waves. In 2019, Hameed et al. [30] introduced a multistage detection method that uses piezoelectric lead zirconate titanate (PZT) transducers to excite and sense the Lamb wave signals.

In 2020, Knitter-Piatkowska et al. [31] developed the concept and verified the experimental possibility of using a wavelet transform to assess a steel structure’s condition. In 2020, Palacz et al. [32] presented a process for numerical modeling of one-dimensional problems related to the propagation of elastic waves and their application for damage detection purposes.

In 2021, Golub et al. [33] employed a 2-D mathematical model to simulate Lamb wave excitation and sensing via rectangular piezoelectric-wafer active transducers mounted on the surface of an elastic plate with rectangular surface-bonded obstacles (stiffeners) with interface defects.

In 2021, Anand et al. [34] used ultrasonic arrays to detect defects in composite structures employed in the aerospace industry. In 2021, Raisutis et al. [35] proposed the variation in the phase velocity of ultrasonic guided waves as a new criterion for defect detection.

The present study explores the use of piezoelectric ceramic components attached to aluminum alloy plates as plate wave actuators and sensors to actively monitor aluminum alloy plates for circular hole damage and fatigue crack scattering wave identification analysis. Time-frequency domain analysis of transient wave signals was carried out according to wavelet transform [36], the damage location was identified from the signal analysis of the damage scattered wave, and the frequency spectrum characteristics of the plate circular hole damage and the through-thickness cracks scattered wave are discussed. An objective function was set according to the wave time-of-flight of the flexible wave reflected from the edge of the damage zone to each receiving point and the theoretical value of the flexible wave velocity. The location of the circular hole was searched by the simplex algorithm [37].

## 2. Basic Theory

A plate wave is constrained by the free boundaries of the upper and lower surfaces of the plate. Only those waves that resonate in the thickness direction can be transmitted along the plate for any distance. If the resonance cannot be formed in the thickness direction, it quickly attenuates and dissipates. Plate waves with different vibration modes or frequencies in the thickness direction have unequal phase velocities. This phenomenon is called dispersion. This section briefly describes the phase velocity and group velocity (energy velocity) of the Lamb wave propagating in a homogeneous isotropic plate.

### 2.1. Phase Velocity of the Plate Wave

**Figure 1:**
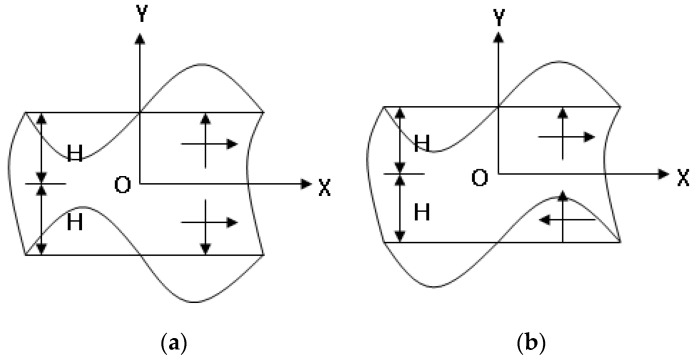
Symmetrical and antisymmetric plate wave displacement. (**a**) Symmetrical plate wave. (**b**) antisymmetric plate wave.

Plate waves are divided into two types according to the symmetry of the wave displacement with respect to the midplane of the plate: anti-symmetric waves and symmetric waves. The former are called flexural waves, and the latter are called extensional waves. Figure 1 is a diagram of symmetric and antisymmetric plate wave displacement.

To simplify the expression, the time-harmonic factor $e^{i\omega t}$ is omitted in the following derivation, and the boundary conditions of the upper and lower surfaces of the plate are:

(1) $$S_{xy}=S_{yy}=0,\;y=\pm H,-\infty <x<\infty$$

The potential functions are assumed to be:

(2a) $$\Phi \left(x,y\right)=F\left(y\right)e^{ikx}$$

(2b) $$\Psi \left(x,y\right)=G\left(y\right)e^{ikx}$$

where *k* is an unknown real wave number, F(y) and G(y) are undetermined functions. The separation of the variables method satisfies the following:

(3a) $$F^{\prime \prime }\left(y\right)-\eta_{1}^{2}F\left(y\right)=0$$

(3b) $$G^{\prime \prime }\left(y\right)-\eta_{2}^{2}G\left(y\right)=0$$

where $\eta_{j}=\sqrt{k^{2}-k_{j}^{2}}$. Inside the plate (−H<y<H), the general solution of the above equation is

(4a) $$F\left(y\right)=Asinh(\eta_{1}y)+Bcosh(\eta_{1}y)$$

(4b) $$G\left(y\right)=Csinh(\eta_{2}y)+Dcosh(\eta_{2}y)$$

where *A*, *B*, *C* and *D* are constants. When B=C=0, the displacement field assumes the following form:

(5a) $$U\left(x,y\right)=\left[ikAsinh\left(\eta_{1}y\right)-\eta_{2}Dsinh\left(\eta_{2}y\right)\right]e^{ikx}$$

(5b) $$V\left(x,y\right)=\left[\eta_{1}Acosh\left(\eta_{1}y\right)+ikDcosh\left(\eta_{2}y\right)\right]e^{ikx}$$

The wave propagation behavior of the antisymmetric plate wave can be obtained from Equation (5a,b). According to the conditions for the existence of nonzero solutions of Equation (5a,b), the dispersion equation can be obtained as

(6) $$\frac{tanh(\eta_{2}H)}{tanh(\eta_{1}H)}=\frac{{\left(2k^{2}-k_{2}^{2}\right)}^{2}}{4k^{2}\eta_{1}\eta_{2}}$$

The solution of the above formula is the wave number *k*; then, the wave velocity *c* can be calculated by k=ω/c.

If A=D=0, the displacement field is expressed as

(7a) $$U\left(x,y\right)=\left[ikBcosh\left(\eta_{1}y\right)-\eta_{2}Ccosh\left(\eta_{2}y\right)\right]e^{ikx}$$

(7b) $$V\left(x,y\right)=\left[\eta_{1}Bsinh\left(\eta_{1}y\right)+ikCsinh\left(\eta_{2}y\right)\right]e^{ikx}$$

This plate wave motion is symmetrical to the *x*-axis. If there is a nonzero solution in Equation (7a,b), *k* must satisfy the following dispersion equation:

(8) $$\frac{tanh(\eta_{1}H)}{tanh(\eta_{2}H)}=\frac{{\left(2k^{2}-k_{2}^{2}\right)}^{2}}{4k^{2}\eta_{1}\eta_{2}}$$

### 2.2. Group Velocity of the Plate Wave

**Figure 2:**
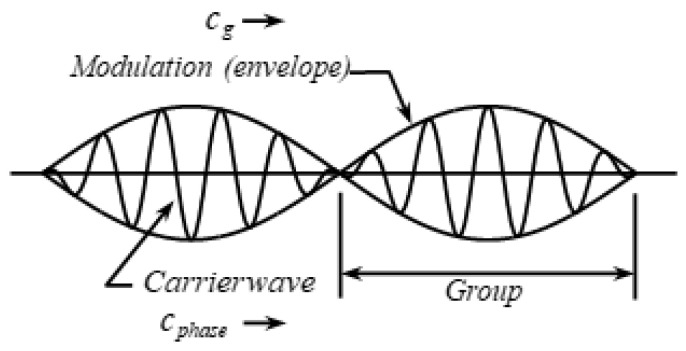
Diagram of wave group propagation.

The plate waves usually seen are not generated by monochromatic sources. If one considers a group of waves with the same amplitude but slightly different frequency and wave-number, and the forward wave propagates along the *x*-axis, the displacement of the composite wave can be described by the following mathematical formula:

(9) $$u=A\left\{sin(k_{1}x-\omega_{1}t)+sin(k_{2}x-\omega_{2}t)\right\}=Csin\left(\overset{-}{k}x-\overset{-}{\omega }t\right)$$

where the modulation amplitude C=2Acos(δk⋅x−δω⋅t), $\delta k=\left(k_{1}-k_{2}\right)/2$, and $\delta \omega =\left(\omega_{1}-\omega_{2}\right)/2$. The frequency of the carrier wave is $\overset{-}{\omega }=\left(\omega_{1}+\omega_{2}\right)/2$, and its wave-number is $\overset{-}{k}=\left(k_{1}+k_{2}\right)/2$. As shown in Figure 2, the modulation amplitude C(x, t) of the entire group wave is the envelope of the carrier wave. The velocity of the wave group is called group velocity, also known as energy velocity. The group velocity $c_{g}$ of the wave group is

(10) $$c_{g}=\underset{\delta k\to 0}{\ell im}\frac{\delta \omega }{\delta k}=\frac{d\omega }{dk}$$

For plate waves, the phase velocity has dispersion characteristics, and the phase velocity, angular frequency ω, and wave vector k are implicit functions. From the dispersion Equations (6) and (8), the group velocities of the antisymmetric plate wave and the symmetric plate wave can be calculated as follows:

(11) $$c_{g}=-\frac{\partial \Omega /\partial k}{\partial \Omega /\partial \omega }$$

where the characteristic function Ω(k, ω) of the antisymmetric plate wave is

(12) $$\Omega \left(k,\;\omega \right)=4k^{2}\eta_{1}\eta_{2}tanh(\eta_{2}H)-\left(2k^{2}-k_{2}^{2}{)}^{2}tanh(\eta_{1}H\right)$$

and the characteristic function Ω(k, ω) of the symmetric plate wave is

(13) $$\Omega \left(k,\;\omega \right)=4k^{2}\eta_{1}\eta_{2}tanh(\eta_{1}H)-\left(2k^{2}-k_{2}^{2}{)}^{2}tanh(\eta_{2}H\right)$$

The speed of a plate wave is a function of frequency or wavelength. According to the resonance mode of the plate wave in the thickness direction, many phase velocity and group velocity dispersion curves can be measured.

**Figure 3:**
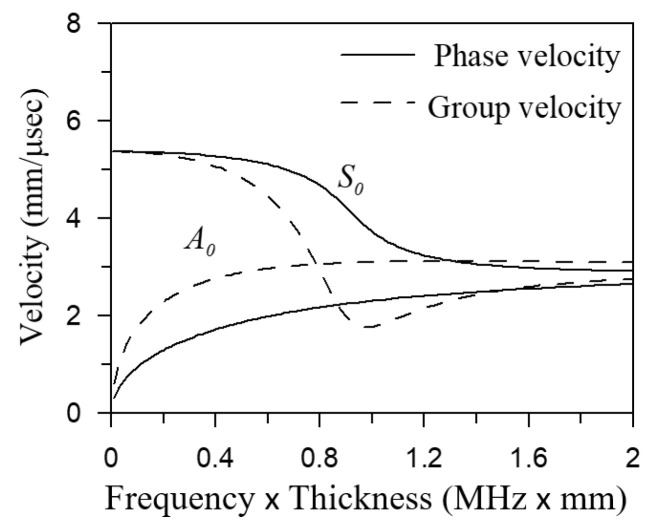
Dispersion curves.

Figure 3 shows the dispersion curves of the phase velocity and group velocity of the fundamental antisymmetric $A_{0}$ and fundamental symmetric $S_{0}$ plate waves of an aluminum alloy plate. Generally speaking, the lower-frequency plate wave is easier to excite, and the amplitude of the plate wave is more obvious. In this study, the excited plate wave was selected as the $A_{0}$ mode.

## 3. Experiments and Measurements

### 3.1. Experimental Architecture Setup

**Figure 4:**
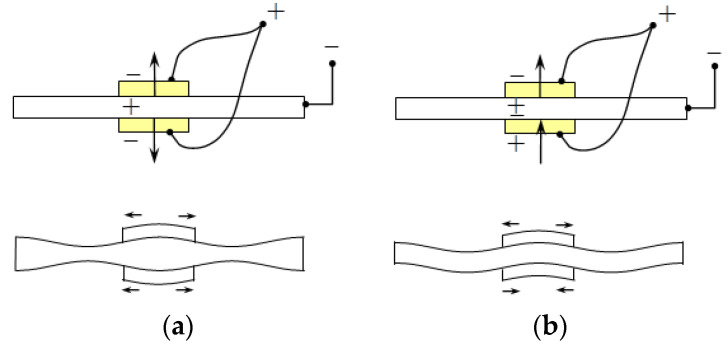
Actuation and generation of symmetric and antisymmetric plate waves. (**a**) Symmetric, (**b**) Antisymmetric.

**Figure 5:**
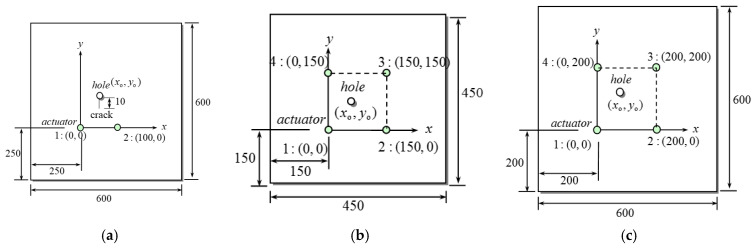
Three types of test plates. (unit: mm). (**a**) Type #1, (**b**) type #2, (**c**) type #3.

An aluminum alloy plate with a thickness of 1 mm and number 6061 was used as the test plate for the experiment in this study. The material parameters of the number 6061 aluminum alloy plate are listed in Table 1. To simultaneously discuss the influence of the size effect of the test plate on damage detection, two sizes of test plates were selected, respectively 450 × 450 mm and 600 × 600 mm. The method of generating a plate wave is shown in Figure 4. Circular piezoelectric ceramic (PZT-4) discs with a diameter of 6.35 mm and a thickness of 0.254 mm were used as the actuator and sensor for plate wave generation. The PZT-4 disc was polarized in the vertical direction, and the charge coefficients were d_33_ = 289 pC/N, d_31_ = −123 pC/N. The layout of the three types of experiments designed in this study is shown in Figure 5.

The first type of test plate is shown in Figure 5a. Two pairs of PZT-4 discs are pasted 250 mm from the edge of the plate. Each pair of actuators is composed of two PZT-4 discs, which are respectively pasted on the upper and lower surfaces of the same position of the plate with conductive epoxy, and the polarization directions of the upper and lower PZT discs are arranged in anti-symmetrical placement (refer to Figure 4b). The PZT-4 discs of each set are separated by 100 mm, and the circular hole damage forms an equilateral triangle with the two sets of PZT-4 discs. Acrylic strips are attached to the four edges of the test plate to reduce the influence of edge reflection. There is a crack with a length of 10 mm beginning at (50, 81.6) mm and ending at the center of the circular hole, and the influence of the circular hole and the crack tip on the scattered plate wave is discussed.

The second type of test plate is shown in Figure 5b. There are four pairs of PZT-4 discs from a 150 × 150 mm square array on the upper and lower surfaces of the 450 × 450 mm plate, each at a distance of 150 mm from two edges of the plate.

The third type of test plate is shown in Figure 5c. This test plate is similar to and slightly larger than the second test plate: the plate is 600 × 600 mm. The circular PZT-4 discs are pasted at a distance of 200 mm from two edges of the plate, and the discs of each set are separated by 200 mm to form a square array. The larger size of the test plate can delay the time-of-flight of the plate wave reflected by the plate edge and can also separate the scattered wave of the circular hole damage and the plate wave group of the direct wave transmitted to the sensor.

**Figure 6:**
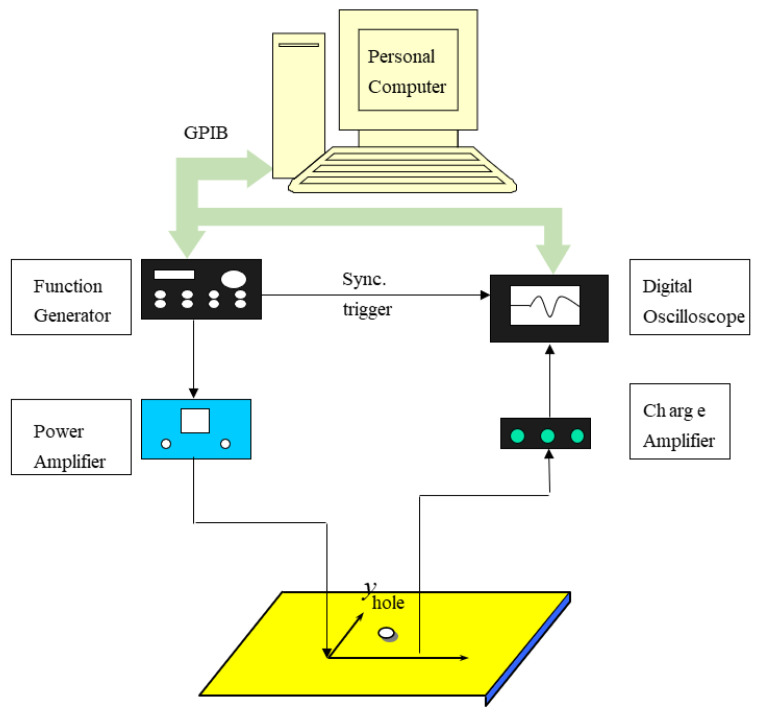
Experimental setup of the active damage detection.

Figure 6 shows the diagram of the experimental device for detecting the damage position of the aluminum plate.

**Figure 7:**
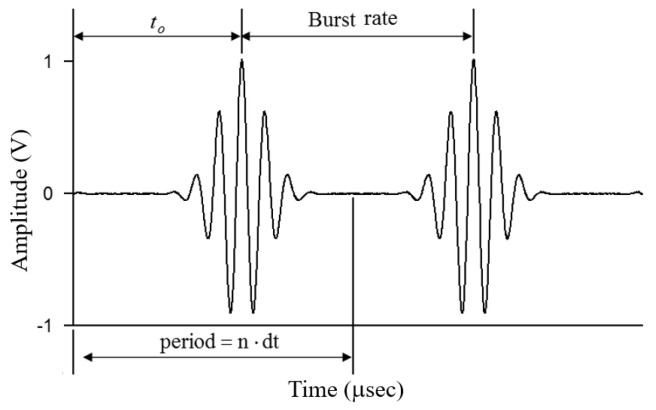
Gaussian cosine pulse function.

In the experiments of the first, second, and third types of test plates, the Gaussian cosine pulse function calculated by the personal computer was transmitted to an HP33250A function generator (Agilent Technologies, Santa Clara, CA, USA) through an NI-GPIB interface card (National Instruments, Austin, TX, USA). The Gaussian cosine pulse function is

(14) $$f\left(t\right)=cos[\omega_{o}\left(t-t_{o}\right)]exp\left[-0.5{\left(\frac{t-t_{o}}{T_{o}}\right)}^{2}\right]$$

where $T_{o}$ is the period of the Gaussian cosine pulse wave, $\omega_{o}$ is the angular frequency of the pulse wave, and $t_{o}$ is the time corresponding to the peak of the pulse wave. The diagram of Gaussian cosine pulse function is detailed in Figure 7, where *n* is the total number of sampling points, dt represents the sampling time (unit: μsec), period represents the appearance time of the pulse wave, and burst rate is the intermittent time of pulse wave generation.

To prevent the piezoelectric actuator from continuously driving and overheating, and to reserve enough time for the residual wave reflected by the plate edge to dissipate, the function generator was made to intermittently generate a Gaussian cosine pulse every 1 second with a maximum amplitude of 50–75 mV. The Gaussian cosine pulse signal was input to a power amplifier ENI 325LA (Bell Electronics Inc., Washington, DC, USA), which amplified the maximum amplitude of the Gaussian cosine pulse to 15.8–23.7 V to drive the PZT-4 discs attached to the surface of the aluminum alloy plate to excite the plate wave. The plate wave signal was received by another pair of PZT-4 discs that were connected to a charge amplifier Vallen AP-1 (Vallen Co., Santa Fe Springs, CA, USA) to amplify the signal. The plate wave signals were captured and stored by a digital oscilloscope.

**Figure 8:**
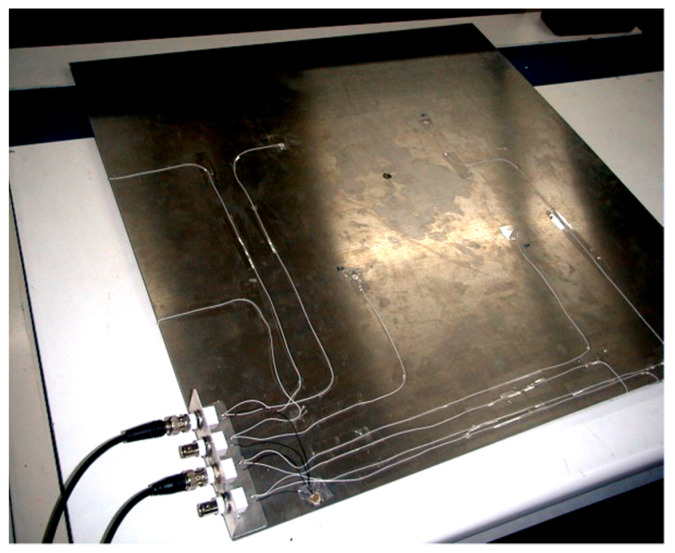
**The** aluminum alloy test plate.

**Figure 9:**
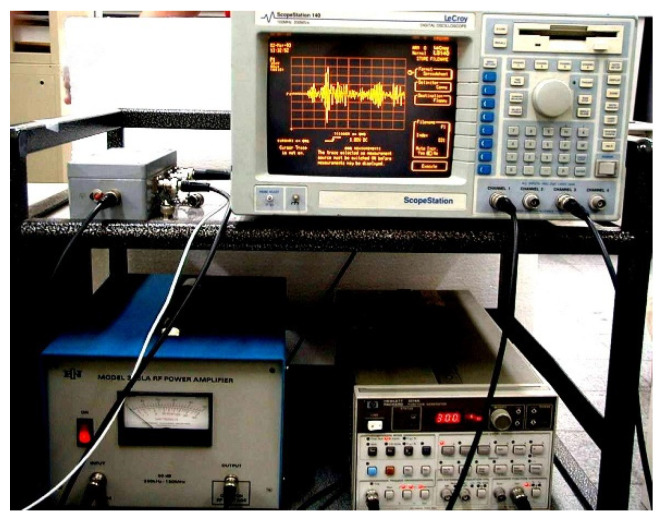
The experimental equipment.

Figure 8 and Figure 9 are the photographs of the aluminum alloy test plate with a circular hole and the equipment used in the experiment.

### 3.2. Time Domain and Frequency Domain Analysis of High-Frequency Signals

The flexural wave generated by the circular PZT-4 disc on the plate was a dispersive wave, the wave speed of each frequency component was not equal, and the waveform changed with the change of the wave propagation distance. Application of wavelet transform analysis could measure and analyze the group velocity of dispersion waves. When the wavelength of the plate wave was proportional to the characteristic length of the plate damage, the natural vibration mode of the plate damage was excited. Therefore, the energy of the incident wave would be absorbed by the damage of the plate so that the amplitude of the scattered wave diffracted from the edge of the damage was sharply reduced, which constituted the spectral characteristic of the scattered wave of the damage.

#### 3.2.1. Wavelet Transform Analysis

Wavelet transform is a widely used time-frequency domain analysis method suitable for the expansion of nonstationary continuous functions. The mother wavelet function is orthogonal and is the kernel function that constitutes the wavelet transformation. It performs a generalized cross-correlation operation with the transient signal, which can extract components similar to the mother wavelet in the signal and has different resolutions in different frequency domains. Continuous wavelet transform can be expressed as follows:

(15) $$CWT\left(a,\;b\right)=\frac{1}{\sqrt{a}}\overset{\infty }{\underset{0}{\int }}f\left(t\right)\Psi^{*}\left(\frac{t-b}{a}\right)\;dt$$

where Ψ(t) is the mother wavelet function, the superscript * represents the complex conjugate, the parameter a represents the scaling factor of the time variable, and the parameter b represents the time delay. When a decreases, the period of the mother wavelet shrinks. Conversely, when a increases, the period of the mother wavelet enlarges. Therefore, a can be used as a trade-off parameter between the time resolution and the frequency resolution, and the wavelet transform is used to map the transient signal in the time domain to the time-frequency domain.

In the physical sense, the wavelet transform maps the transient signal *f* to the wavelet transform coefficients of each frequency component, and the value corresponding to the peak of the envelope is the wave time-of-flight for the wave packet of that frequency to reach the receiving point. If the transient signal is a plate wave signal, the product of the wave time-of-flight difference of the wave packet and the plate wave group velocity is the wave propagation distance of the plate wave energy. Therefore, the group velocity of the plate wave is also called the energy velocity of the plate wave.

**Figure 10:**
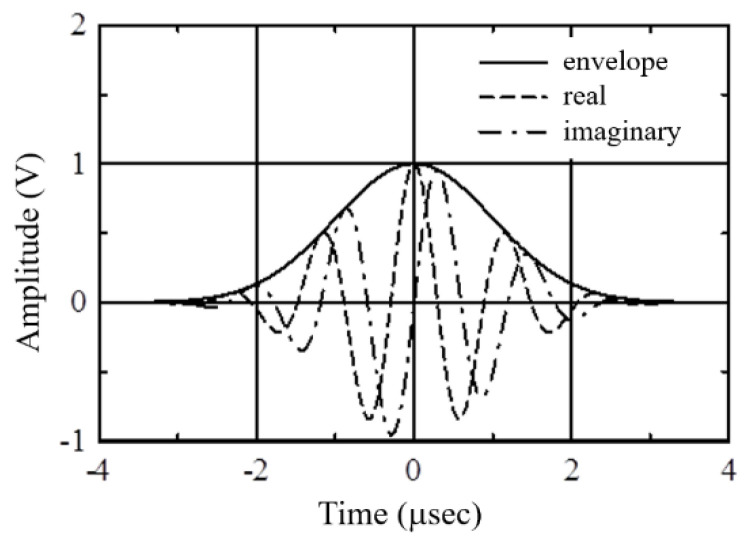
Gaussian pulse mother wavelet function.

This study used Gaussian pulse as the mother wavelet function, and its mathematical formula is as follows:

(16) $$\Psi \left(t\right)=e^{i\omega_{o}t}e^{-t^{2}/2}$$

where $\omega_{o}=5.3\times 10^{6}\mathrm{rad}/\sec$. Figure 10 shows the real part, imaginary part, and envelope of the Gaussian pulse function. According to Equation (16), the envelope is the absolute value of the Gaussian pulse wave function, which is the Gaussian distribution function. Because the real and imaginary parts of the Gaussian pulse wave function are both oscillating functions, it is difficult to identify the peak value of the wavelet transform coefficient and its corresponding time delay. Therefore, the time delay corresponding to the peak value of the envelope of the wavelet transform coefficient is usually used as the target for calculating the wave time-of-flight difference of the transient signal.

#### 3.2.2. Measurement of Group Velocity

Wavelet transform analysis can be used to measure and analyze the group velocity of dispersive waves by extending a straight line from the wave source of the transient wave and selecting any two field points on the straight line to acquire the transient wave signal. Wavelet transform processing can then be used to calculate the time delay of the peak of the envelope of the wave packet at different frequencies. The time delay difference Δt of the two sets of signals is the wave time-of-flight required for the wave packet to pass the receiving point field distance difference Δx, and the measured value of the dispersion wave group velocity is

(17) $$C_{g}\left(\omega \right)=\Delta x/\Delta t$$

**Figure 11:**
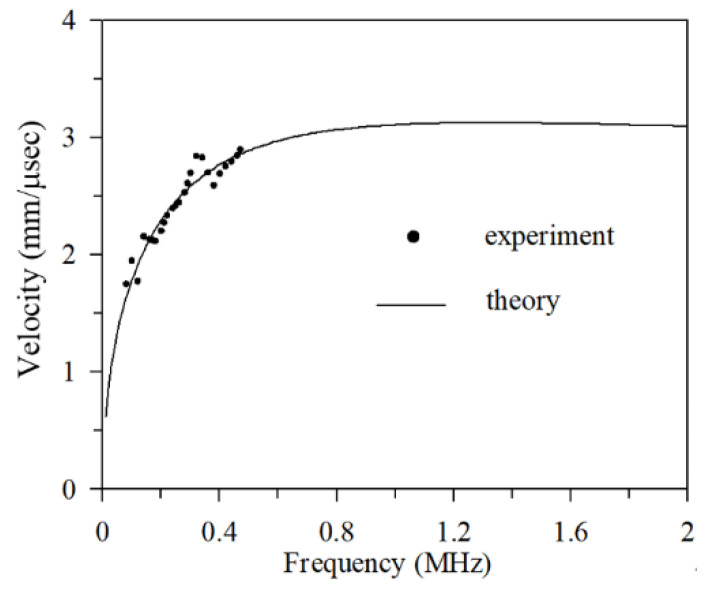
Group velocity of the $A_{0}$ plate wave. The solid line is the theoretical value; the dots are experimental value.

In test plate #1, the PZT-4 disc located at (100, 0) mm was used as the plate wave transmitter. A Deci SE1000H acoustic emission transducer (KRN Services Inc., Grandville, MI, USA) was used to receive the signal at (170, 0) mm and (200, 0) mm, and the receiving points are 30 mm apart. A higher sampling frequency can obtain a better measurement value of energy speed. The sampling frequency in this experiment was 25 MHz, and the number of sampling points was 8064. Figure 11 shows the comparison between the theoretical value and the experimental value.

#### 3.2.3. Spectra Characteristic of the Dispersion Plate Wave

In addition to the scattered wave signal, the plate wave signal of the plate with circular hole damage also includes the plate wave signal of direct wave propagation. Under ideal conditions, the scattered wave signal of the circular hole can be obtained if the plate wave signal of the defective plate in the same path is subtracted from the plate wave signal of the nondefective plate.

**Figure 12:**
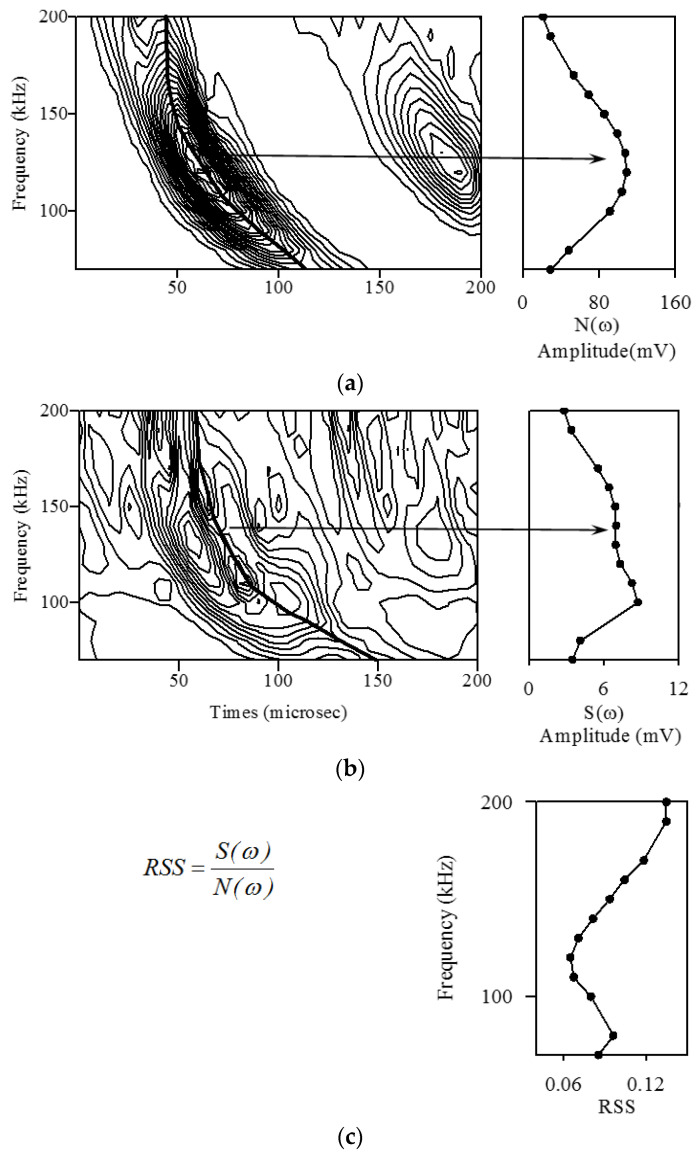
Spectra characteristic of the dispersion plate wave. (**a**) Sensor Spectrogram, (**b**) scatter spectrogram, (**c**) relative scatter spectrum.

Figure 12a shows the contour map of the wavelet transform coefficient envelope of the direct wave transmission signal between piezoelectric transducers. The spectrogram on the right is the response N(ω) of the peak value of the wavelet transform coefficient envelope ridgeline to the frequency of the plate wave. The contour map shown in Figure 12b is the envelope of the wavelet transform coefficient of the circular hole scattered wave, and the spectrum on the right is the response S(ω) of the peak of the ridgeline with respect to the frequency.

The quotient of dividing S(ω) by N(ω) is called the relative scatter spectrogram (*RSS*) as follows:

(18) $$RSS=\frac{S\left(\omega \right)}{N\left(\omega \right)}$$

RSS is determined by subtracting the influence factor of the plate wave generation and measurement system on the plate wave response of different frequencies to obtain the true spectrum characteristics of the scattered wave, as shown in Figure 12c.

Because the amplitude of the circular hole scattered wave is very small after the above two sets of signals are subtracted, there is a group of waves with an amplitude equal to or slightly smaller than that of the scattered wave near the arrival time of the direct wave propagating wave packet. The contour map of the envelope of the scattered wave wavelet transform coefficient in Figure 12b shows other peaks near the main ridge, but those peaks have nothing to do with the scattered wave signal.

### 3.3. Inverse Calculation Model of Circular Hole Damage Position

The wave propagation path of the plate wave scattered from the circular hole was different from the wave propagation path of the plate wave directly transmitted between the PZT-4 discs. The signal transmission and reception between each pair of PZT-4 discs resulted in a set of wave time-of-flight difference data.

There were two sets of PZT-4 discs on the first and second types of experimental test plates. The discs sent to and received from each other one by one, and the wave time-of-flight difference of six groups (1 to 2, 1 to 3,1 to 4, 2 to 3, 2 to 4, 3 to 4) of related damage scattered waves were obtained.

An objective function *Q* can be defined as the square of the difference between the possible wave propagation path $d_{trial}$ and the measured wave propagation path $d_{measured}$ as follows:

(19) $$Q=\overset{M}{\underset{i=1}{\sum }}{\left(d_{trial}-d_{measured}\right)}_{i}^{2}$$

where *M* is the total number of data points for different wave propagation paths or plate wave frequencies.

Plate wave propagation is inherently dispersive, and plate waves of different frequencies have unequal phase velocities and group velocities. The plate wave signal is used to calculate the damage position, and the product of the group velocity and the wave path time difference is usually used as the measured wave path difference $d_{measured}$. The scattered wave signal of the plate wave was very small, after a long wave propagation in the absence of other wave phase information comparison with approximated wave paths. Therefore, a more suitable way to measure the wave time-of-flight of a plate wave is to use the maximum peak of the plate wave signal envelope as an index: the wave propagation time of the plate wave packet.

Equation (19) can be slightly modified into the following:

(20) $$Q=\overset{M}{\underset{i=1}{\sum }}{\left[\Delta x_{1}+\Delta x_{2}-\Delta x_{3}-2R-c_{g}\left(\omega \right)\cdot \Delta t_{measured}\right]}_{i}^{2}$$

(21a) $$\Delta x_{1}=\sqrt{{\left(x_{o}-x_{1}\right)}^{2}+{\left(y_{o}-y_{1}\right)}^{2}}$$

(21b) $$\Delta x_{2}=\sqrt{{\left(x_{o}-x_{2}\right)}^{2}+{\left(y_{o}-y_{2}\right)}^{2}}$$

(21c) $$\Delta x_{3}=\sqrt{{\left(x_{1}-x_{2}\right)}^{2}+{\left(y_{1}-y_{2}\right)}^{2}}$$

where $\Delta x_{1}$ is the distance from the piezoelectric actuator $\left(x_{1},y_{1}\right)$ to the center of the hole damage, $\Delta x_{2}$ is the distance from the center of the hole damage to the piezoelectric sensor $\left(x_{2},y_{2}\right)$, $\Delta x_{3}$ is the linear distance from the piezoelectric actuator to the sensor, $c_{g}\left(\omega \right)$ is the plate wave group velocity corresponding to the angular frequency ω, $\Delta t_{measured}$ is the measured value of the wave path time difference between the diffracted wave and the direct wave propagating plate wave, and R is the radius of the hole.

**Figure 13:**
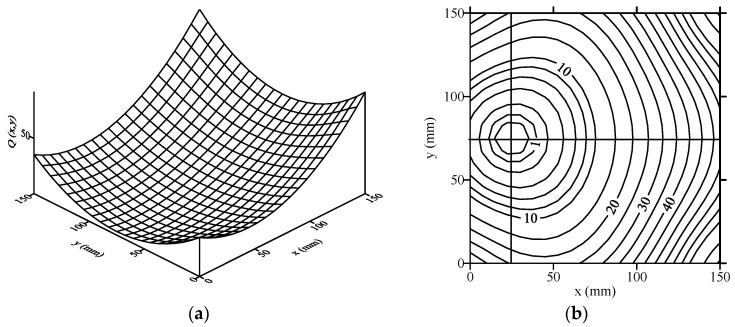
Perspective view and contour map of the objective function for crack location optimization. (**a**) Perspective view, (**b**) contour map.

The objective function described by Equation (20) is a two-dimensional surface with a single global minimum, and the $\left(x_{o},y_{o}\right)$ coordinate corresponding to the global minimum is the center position of the hole defect. Figure 13 shows the perspective view of the two-dimensional surface and its corresponding contour map of the objective function of Equation (20) when R=0.

In this study, the simplex method was used as the optimization procedure to search for the minimum value of the objective function of Equation (20). Generally, when searching for the minimum value of a nonlinear objective function with the least square difference method, the objective function must use local linearization first and then an iteration operation, which requires a large amount of calculation. If the simplex method is used to search for the minimum value, the calculation time of the local linearization of the objective function can be reduced, and the searching procedure is faster than the least square difference method.

An N-dimensional simplex body is constructed by connecting N+1 vertices and the surrounding polygons. The two parameters searched in this study are the center coordinates $\left(x_{o},y_{o}\right)$ and radius R of the hole damage, so the simplex body established is a tetrahedron with four vertices. First, a set of guessed starting values are given, four vertices of a tetrahedron are established near the starting value, and the objective function value of each vertex is compared to find the set of maximum values. Then this vertex is connected to the center of the corresponding triangle with a straight line. Next, a point with a smaller objective function value and once or twice the length of its extension line is found as a new vertex at the midpoint of this line to replace the original vertex and from a new simplex body. The above steps are repeated. The simplex body will gradually shrink until the simplex body is small enough or the function values of the vertices are very close, and then the optimization procedure is terminated.

## 4. Experimental Results and Discussion

### 4.1. Analysis of Time-of-Flight of Scattered Wave from Circular Hole Damage

**Figure 14:**
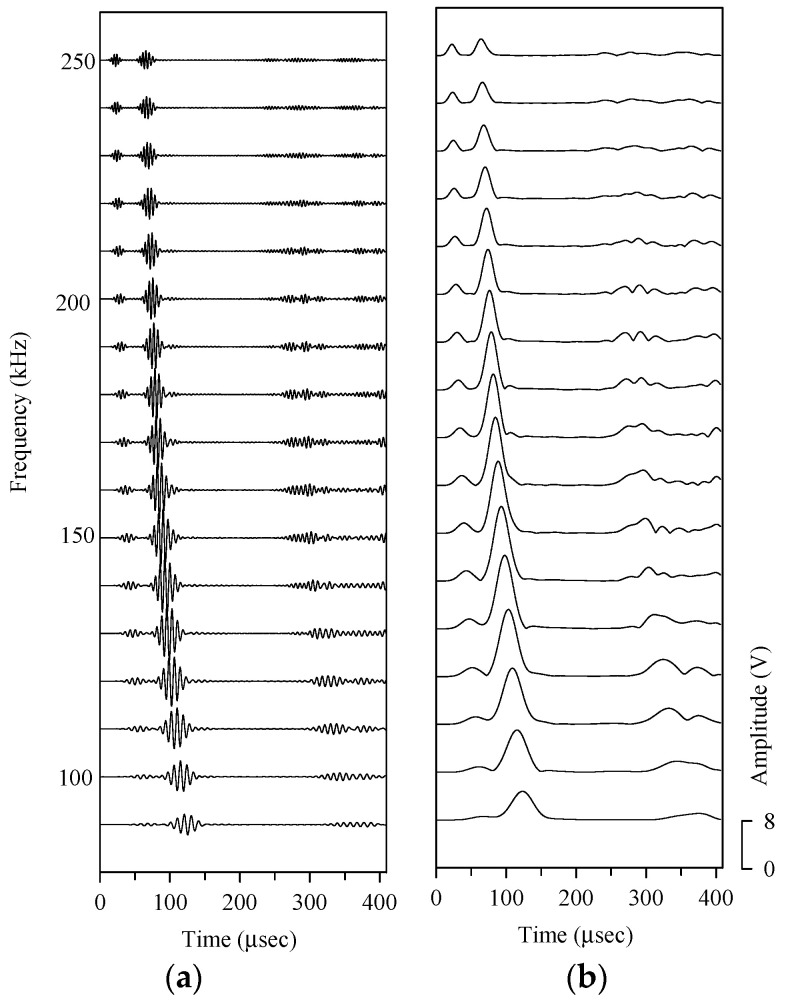
On test plate #1, the wave propagation path of the plate wave from PZT-4 discs 1 to 2 and the envelope after the wavelet transform (without a circular hole). (**a**) Plate wave signal, (**b**) envelope of the plate wave.

**Figure 15:**
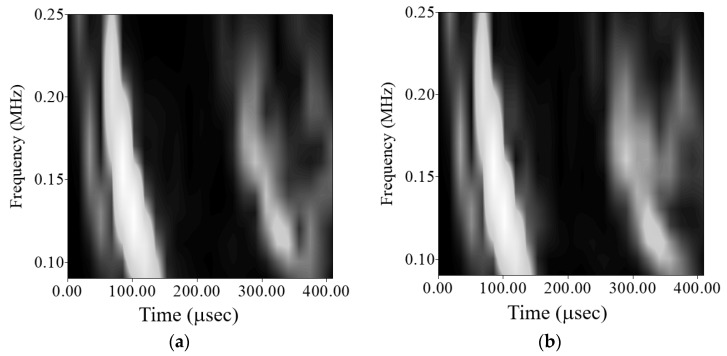

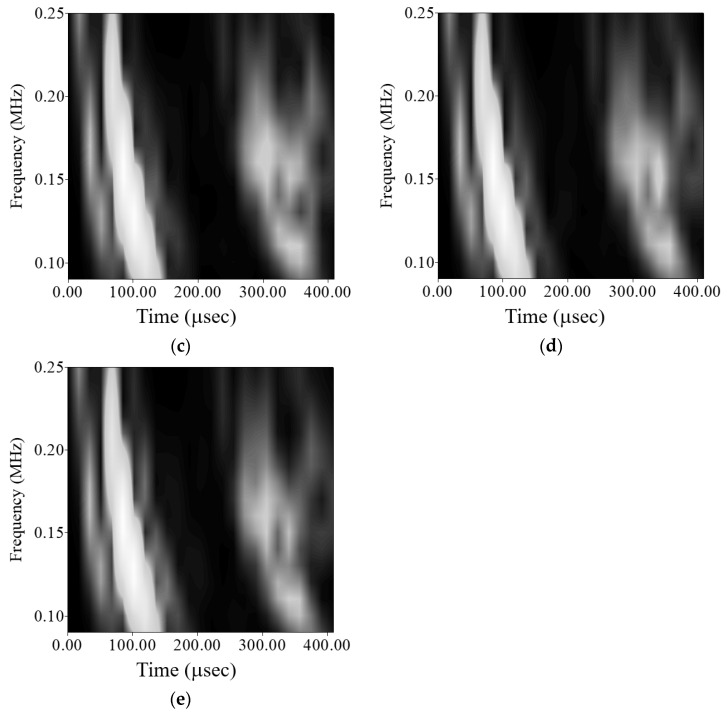
Gray-level diagram of the wave propagation path from PZT-4 discs 1 to 2. (**a**) Without circular hole, (**b**) with a 3-mm-diameter circular hole, (**c**) with a 5-mm-diameter circular hole, (**d**) with an 8-mm-diameter circular hole, (**e**) with a 10-mm-diameter circular hole.

In the first test plate, the plate wave generated by one circular PZT-4 actuator adhered to the surface of the aluminum plate was scattered in all directions, and the plate wave signal is received by another pair of circular PZT-4 sensors. Figure 14 shows the plate wave signals and wavelet transform coefficients of PZT-4 discs 1 to 2. The plate wave signal with a frequency of 90–250 kHz shows that the signal arriving at the position of the PZT-4 sensor between 50–150 μsec belongs to the *A*_0_ anti-symmetric plate wave. In addition to the scattered wave signal, the plate wave signal of the test plate with circular hole damage also includes the plate wave signal of direct wave propagation. The tiny signal immediately behind the *A*_0_ plate wave is the plate wave scattered from the circular hole is the wave that arrives at the PZT-4 sensor between 120–200 μsec. The gray-level diagram after wavelet transform is shown in Figure 15, and the scattering plate wave immediately after the *A*_0_ plate wave can be observed more clearly.

**Figure 16:**
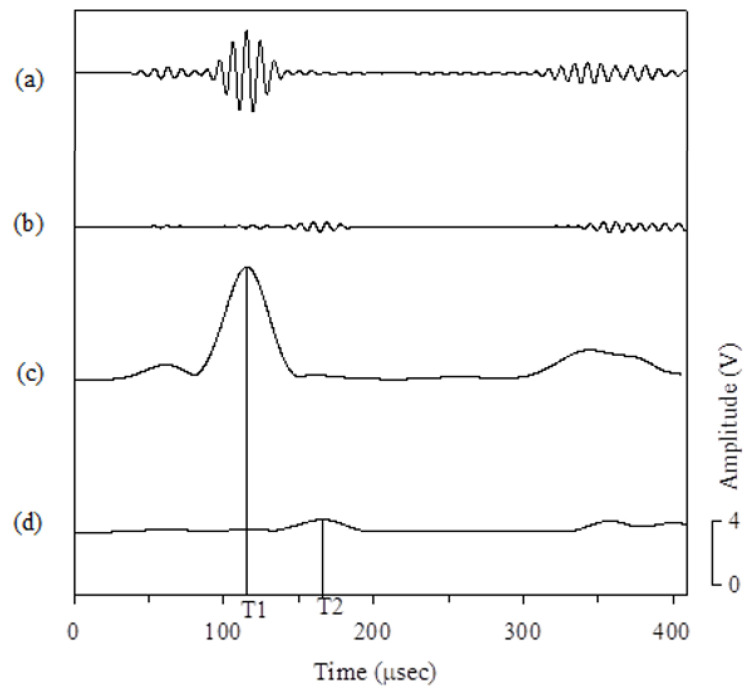
Signal processing of scattered waves from circular hole damage (T1 = 115.4 μsec, T2 = 165.8 μsec). (**a**) The *A*_0_ plate wave transmitted directly to the sensor. (**b**) The *A*_0_ plate wave generated by the PZT-4 actuator scattered by a circular hole with a diameter of 10 mm and then arriving at the PZT-4 sensor. (**c**) The envelope of (**a**). (**d**) The envelope of (**b**).

Under ideal conditions, the scattered wave signal of the circular hole can be obtained if the plate wave signal of the damaged plate in the same path is subtracted from the plate wave signal of the undamaged plate. The numbers in Figure 16 represent the time-of-flight of the envelope wave peak. Figure 16a shows the *A*_0_ plate wave transmitted directly to the sensor, and the Figure 16b shows the *A*_0_ plate wave generated by the PZT-4 actuator that is scattered by a circular hole with a diameter of 10 mm and then arrives at the PZT-4 sensor. The Figure 16c shows the envelope of Figure 16a, and the signal peak of the time-of-flight corresponding to the envelope peak is 115.4 μsec. Figure 16d shows the envelope of Figure 16b, and the time-of-flight corresponding to this peak is 165.8 μsec. The time-of-flight difference between the two discs is 50.4 μsec. The group velocity of the plate wave with a frequency of 100 kHz is 1.7775 mm/μsec, so the wave path difference between the direct wave propagation and the plate wave scattered by the circular hole is 89.59 mm.

To verify the time-of-flight difference between the direct wave propagation and the scattered plate wave using the shortest distance of the *A*_0_ plate wave propagation as the criterion, the reflection points $\left(x_{r},\;y_{r}\right)$ of the *A*_0_ scattering plate wave on a 10-mm-diameter circular hole was calculated. If the center of the circular hole is at (50, 86.6) mm, the reflection point falls on the edge of the 10-mm-diameter circular hole, and the coordinates of the reflection point can be expressed as

(22) $$x_{r}=50+5cos\theta ,\;y_{r}=86.6+5sin\theta$$

The wave displacement d of the *A*_0_ plate wave from the source point (which is also the coordinate origin) to the reflection point and then to the PZT-4 sensor is

(23) $$d=\sqrt{x^{2}+y^{2}}+\sqrt{{\left(x_{r}-100\right)}^{2}+y_{r}^{2}}$$

Substituting Equation (22) into (23) and taking the differentiation of θ shows that the minimum value occurs at $\theta =270^{o}$, that is, the reflection point of the shortest wave displacement is located at $\left(x_{r},\;y_{r}\right)=\left(50,\;81.6\right)$ mm, and d=191.4 mm.

This study used the shortest wave propagation distance and the wave path difference of direct wave propagation as the basis for the time-of-flight difference. The results show that the maximum error did not exceed ±7%. These data also verify the accuracy of the low-frequency plate wave in detecting the damage position of the plate.

### 4.2. Analysis of Time-of-Flight of Diffraction Wave from Crack Damage

In test plate #1, an artificial crack with a length of 10 mm was made at (50, 81.6) mm at the edge of the 10-mm-diameter circular hole, and the tip of the crack was at (50, 71.6) mm.

**Figure 17:**
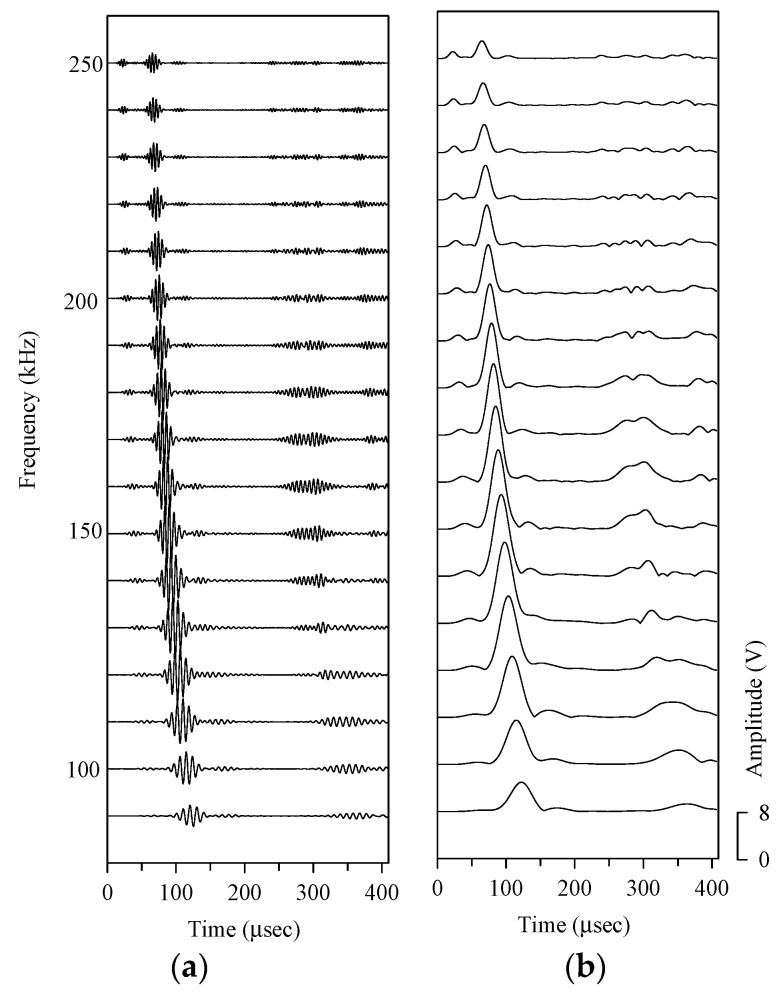
The wave propagation path of the plate wave from PZT-4 discs 1 to 2 and the envelope after the wavelet transform on test plate #1 with a 10-mm-diameter circular hole and a 10 mm crack. (**a**) Wave signal, (**b**) envelope.

**Figure 18:**
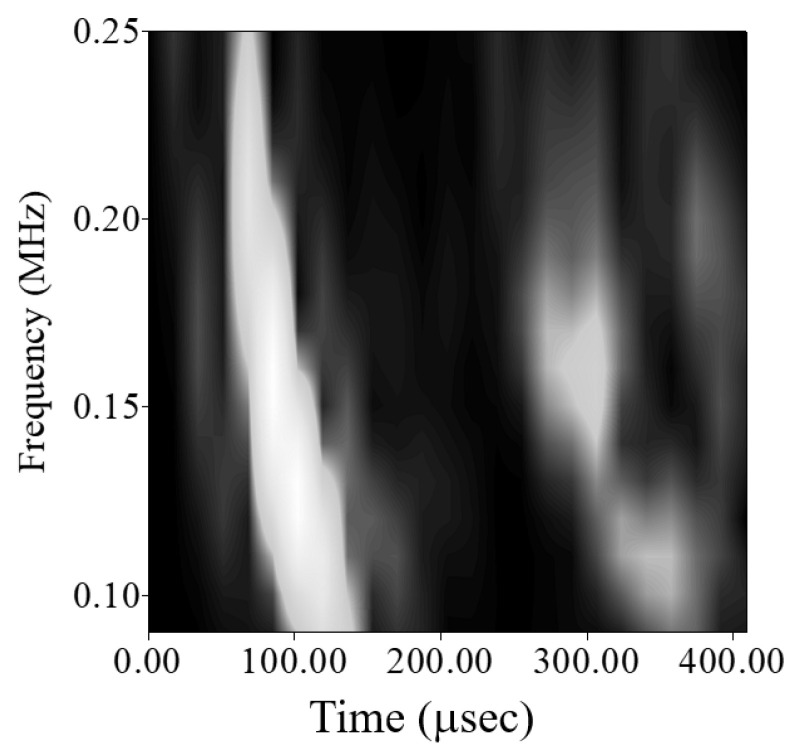
Gray-level diagram of the wave propagation path from PZT-4 discs 1 to 2 on test plate #1 with a 10-mm-diameter circular hole and a 10 mm crack.

Figure 17 shows the plate wave signal and wavelet transform coefficient of the test plate with a 10-mm-diameter circular hole and a crack of 10-mm-long crack. Figure 18 shows the gray-level diagram. The *A*_0_ plate wave generated by the actuator was diffracted by the 10-mm-diameter crack and reached the *A*_0_ plate wave of the piezoelectric sensor. The shortest wave path reflection point was at (50, 71.6) mm, and the shortest wavelength was 174.66 mm. Time-of-flight happened to fall between the direct wave propagated *A*_0_ plate wave and the wave scattered by the circular hole damage.

### 4.3. Identification of the Location of Circular Hole Damage

The wave propagation path of the wave diffracted from the plate hole damage was different from that of the plate wave directly propagated between PZT-4 discs. The signal transmission and reception between each pair of PZT-4 discs can generate a set of data of the time-of-flight differences. Four pairs of PZT-4 discs were installed on test plates #2 and #3, respectively. The transducers sent to and received from each other, and the time-of-flight difference of six sets of related damage diffraction waves, were obtained.

The longer the time difference between the two groups of signals, the better the isolation of the signals. The small oscillations between the two groups of waves, which may have been caused by damage of the PZT-4 disc attached to the plate surface, were not noisy because each group of signals were averaged for 20 groups of signals before being captured. The low-frequency signal had some high-frequency electromagnetic wave interference before 40 μsec, and its frequency was clearly distinguished from the frequency of the plate wave.

In test plate #3, the signal isolation of paths 1 to 2, 3 to 4, 2 to 3, and 1 to 4 were better, and the shortest wave displacements were 277.2 mm, 277.2 mm, 304.8 mm and 250 mm, respectively. Theoretical values of the group velocities of the above four paths, the estimated values of the time-of-flight differences, and the experimental values were substituted into the optimization program of the hole damage position described in the previous section to search for the center coordinates of the hole. The estimated center of the circle of the test plate #3 was (81.89, 100.68) mm, which was about 2 mm different from the actual circle center at (80, 100) mm.

### 4.4. Spectrum Analysis of Scattered Waves from Circular Hole Damage

When the wavelength of the plate wave is proportional to the characteristic length of the plate damage, the natural vibration mode of the plate damage is excited. Therefore, the energy of the incident wave is absorbed by the plate damage so that the amplitude of the plate wave scattered from the edge of the damage is greatly reduced. This is the spectral characteristic of the damage-scattered wave.

**Figure 19:**
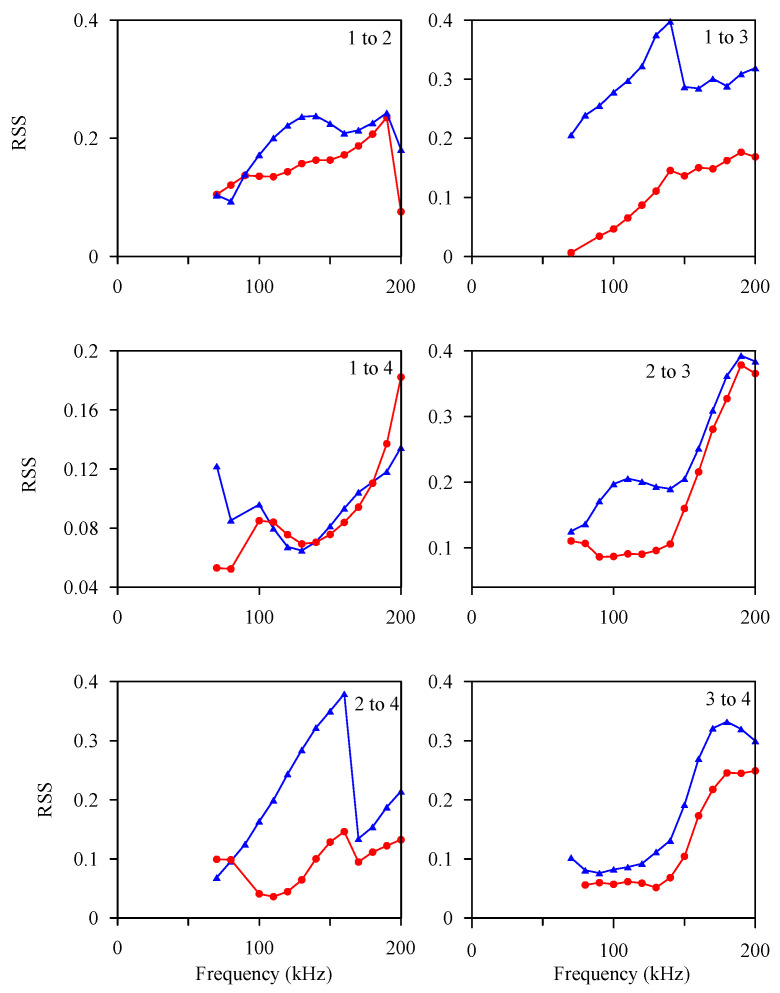
Experimental values of scattered wave spectra for different wave propagation paths on test plate #3. The solid red circle represents a 5-mm-diameter circular hole, and the solid blue triangle represents a 10-mm-diameter circular hole.

Figure 19 shows the experimental values of the spectrum of the scattered wave of the circular hole damage following different wave propagation paths on the 600 × 600 mm test plate #3. The diameters of the circular hole damage are 5 mm and 10 mm, respectively. The relative scatter spectrogram (RSS) had a consistent trend of change in the same wave propagation path, but the RSS spectrum of different wave propagation paths still had some differences. The RSS spectra of paths 1 to 4, 2 to 3, and 3 to 4 all had the characteristic of a notch around 100–125 kHz. The physical meaning of this notch is that near this frequency, the plate wave resonated with the circular hole, and most of the plate wave energy was confined around the circular hole. Only a small part of the wave energy was taken away by the scattered waves and detected by the PZT-4 sensor.

**Figure 20:**
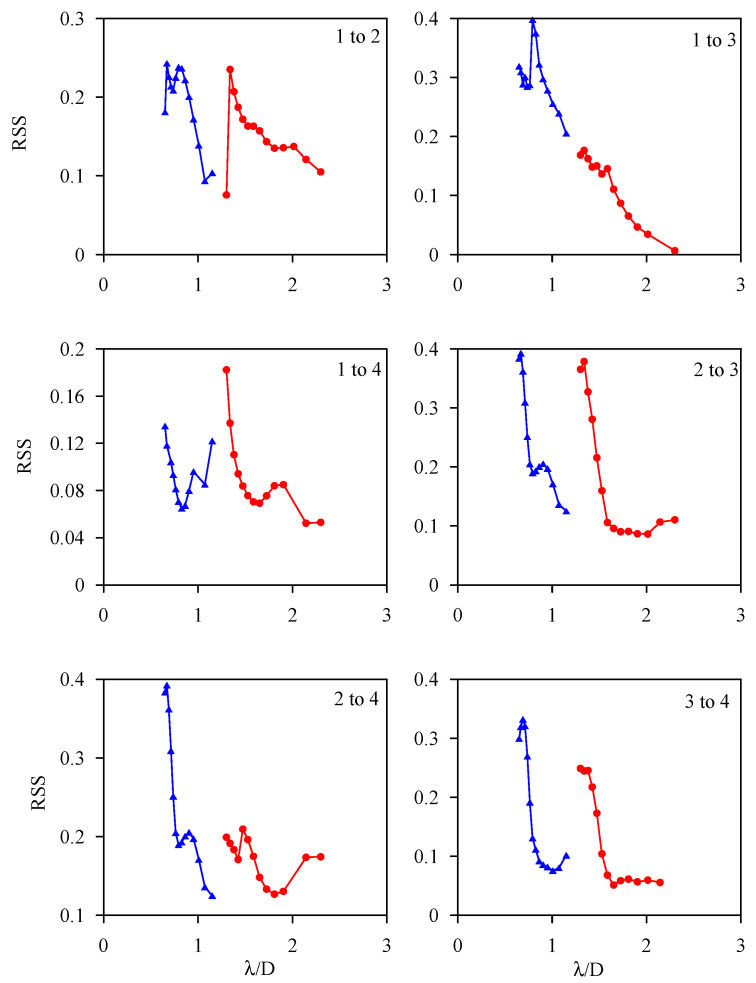
Experimental values of the scattered wave spectrum of different wave propagation paths on test plate #3 with changes to λ/D. The solid red circle represents a 5-mm-diameter circular hole, and a solid blue triangle represents a 10-mm-diameter circular hole.

The frequency at which the plate wave resonates with the hole damage should be related to the size of the hole. Therefore, the RSS curve of each group of scattered waves in Figure 19 was plotted relative to λ/D in Figure 20, where λ is the wavelength of the *A*_0_ plate wave and is also a function of frequency, and D is the diameter of the circular hole damage. Different paths and different circular hole damage sizes had different frequency spectrum characteristics, but the RSS spectrum of circular hole damage diameters of 5 mm and 10 mm had no overlapping area for the λ/D curve. In the future, circular hole damage with a diameter between 5 and 10 mm can be added to establish a complete plate wave spectrum.

## 5. Conclusions

The purpose of this study was not to develop new ultrasonic nondestructive detection methods or active smart monitoring technologies. Instead, the study explored the technology of plate wave active detection of plate hole damage and crack positions and the working principle of size identification using experimental verification by understanding the development space of relevant theories of scattering plate waves.

PZT-4 discs pasted on aluminum alloy plates were used as actuators and sensors for plate waves, which avoided coupling variation caused by the material and thickness of the coupling fluid. The plate wave signal generated by the piezoelectric actuator was a narrow-frequency signal. The time-frequency domain analysis of the plate wave signal combined with frequency scanning technology and wavelet transform enabled calculation of the time-of-flight and spectral characteristic analysis of the scattered wave signal. The plate wave propagated on the discontinuous material interface (such as the plate or the damage) to produce the wave mode conversion of *A*_0_ and *S*_0_ plate wave, but the complicated wave mode conversion made analysis of the scattered wave signal more difficult.

This study developed a time-frequency domain analysis method based on continuous wavelet transformation and analyzed the plate wave generated by the piezoelectric ceramic actuator and the scattered wave signal on two plates, one with a 5-mm circular hole and the other with a 10-mm circular hole. The calculated time-of-flight difference of different wave packets reaching the receiving transducer position was in good agreement with the theoretical estimation result and could be used as the basis for identifying the position of hole damage.

In this study, two PZT-4 discs were directly adhered to the upper and lower surfaces of the test plate as an actuator for generating *A*_0_ plate waves, and as a sensor for detecting *A*_0_ plate waves. Four pairs of PZT-4 transducers were arranged on each test plate to form an array for structural health inspection. This study established an optimization program based on the simplex algorithm to calculate the damage position of the plate. The objective function for optimization was defined as the sum of squares of the time-of-flight difference between the damage scattered wave and the direct wave propagating plate wave. This function has a unique global minimum that can ensure the accuracy of the damage location calculation and obtain good results in experiments.

Under ideal conditions, the plate wave signal containing defects can be obtained by subtracting the plate wave signal without defects from the plate wave signal scattered by the defect. However, in practical applications, the wires are susceptible to electromagnetic interference, contact oxidation and other factors causing noise in the signal transmitted directly between the actuator and the receiver. It is hoped that this problem can be solved in the future to improve the accuracy of analysis.

## Figures and Tables

**Table 1:** Material parameters of number 6061 aluminum alloy plate.

ρ	μ	E	ν	$c_{1}$	$c_{2}$
$\left(g/{\mathrm{cm}}^{3}\right)$	(GPa)	(GPa)		(mm/μsec)	(mm/μsec)
2.861	27.271	73.039	0.339	6.2578	3.0874
